# Primary papilloma of the proximal ureter in a 13-year old boy: A rare case

**DOI:** 10.1016/j.ijscr.2020.05.016

**Published:** 2020-05-23

**Authors:** Mahtab Rahbar, Keykhosro Mardanpour, Maryam Rahbar, Seyed Javad Nasiri

**Affiliations:** a Iran Medical Science University, Iran; b Tehran Medical Science of University, Iran; c Kermanshah University of Medical Science, Iran

**Keywords:** Papilloma, Ureter, Child

## Abstract

**Introduction:**

Approximately 1 percent of the tumors observed in the upper urinary tract are primary tumors of the ureter. The exact diagnosis is done by a histologic study. Most cases need surgery, which is done by complete resection of the tumoral segment of the ureter.

**Presentation of case:**

This study presents a 13-year-old boy who referred to Ali Asghar pediatric hospital with complaints of right flank pain and hematuria for four months. The ultrasonography revealed moderate-grade hydronephrosis. The retrograde urography confirmed a 1.5 cm lesion with filling defects in the proximal segment of the right ureter. The abdominal CT-scan confirmed a 1.5 cm filling defect lesion with a smooth margin in the right proximal ureter adjacent to the ureteropelvic orifice. On cystoscopy, a sessile mass developed in the proximal portion of right ureter and providing pronounced dilation of the ureter and ipsilateral hydronephrosis. Histopathology examination revealed a benign neoplasm composed of a delicate fibrovascular core covered by normal-appearing urothelium. The patient underwent a successful proximal segment resection of the ureter by surgery.

**Discussion:**

Benign tumors of the ureter are much less frequent than malignant ones. Mostly, they involve the lower third of the ureter. Pain, hematuria, and hydronephrosis are the most clinical signs. The final diagnosis can be established with the histologic examination. The choice treatment in is segmental ureterectomy.

**Conclusion:**

Primary ureteral papilloma is extremely rare in the child. The histopathology study is essential for ruling out malignancy. The recurrence and progression of ureteral papilloma are controversial.

## Highlights

• Primary papilloma of the ureteral proximal end is extremely rare.
• Benign tumors of the ureter are much less frequent than malignant ones.
• The main clinical signs of benign ureteral tumor are hematuria, pain and hydronephrosis.
• The exact diagnosis can be established with histologic study.
• The best noninvasive treatment of benign ureteral mass lesion is a segmental resection of ureter.

## 1 Introduction

Primary ureteral papilloma is extremely rare. Urothelial papilloma is a benign exophytic neoplasm that generally does not progress [1,2]. The progression has reported in an association with immunosuppressive therapy [3]. Urothelial papilloma often encountered as a de novo lesion but may arise as a secondary papilloma [4,5]. Urothelial papilloma comprises about 1% of papillary urothelial neoplasms. Most of the patients are less than 50 years old and even can present in children. The male to female ratio is 1.9:1 [4,6]. The most common locations of Urothelial papilloma are posterior or lateral walls of the bladder adjacent to the ureterovesical orifices and also urethra [[3], [4], [5],^7^,^8^]. Generally, cystoscopy shows a small unifocal papillary or elevated lesion. Histologically, the structure of urothelial papilloma consists of a delicate fibrovascular core covered by proliferating normal-appearing urothelium lacking atypia, including the presence of umbrella cells with elongated or oval nuclei. Papillae show slender with minimal branching [4,5]. The Recurrence rate of ureteral papilloma of the bladder is about 9–31%, but there is no risk for progression to higher-grade tumors. Hematuria is the most common clinical symptoms [6,9]. Our study has been designed based on the SCARE statement guidelines 2018 [10].

## 2 Presentation of case

**Fig. 1:**
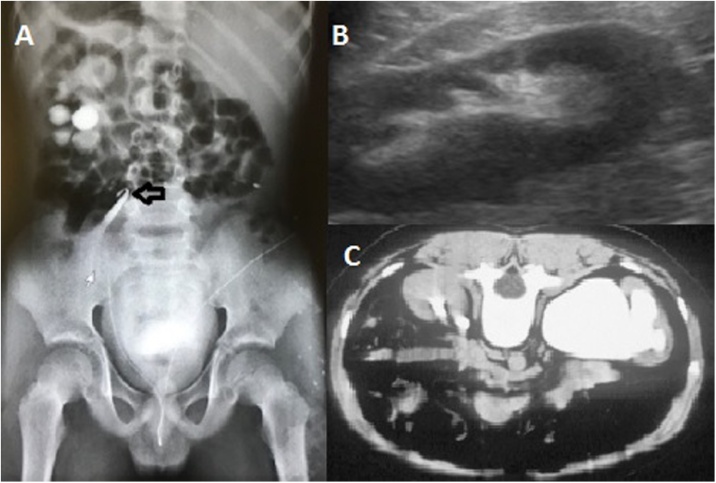
(A) Ultrasonographic showing a moderate left hydronephrosis with thin renal cortex; (B) Retrograde ureteropyelography revealing ureteral papilloma, left upper-ureteral filling defect of the ureter; (C) Abdominal computed tomography scan confirmed a filling defect lesion with smooth margin in the proximal left ureter and left hydronephrosis.

**Fig. 2:**
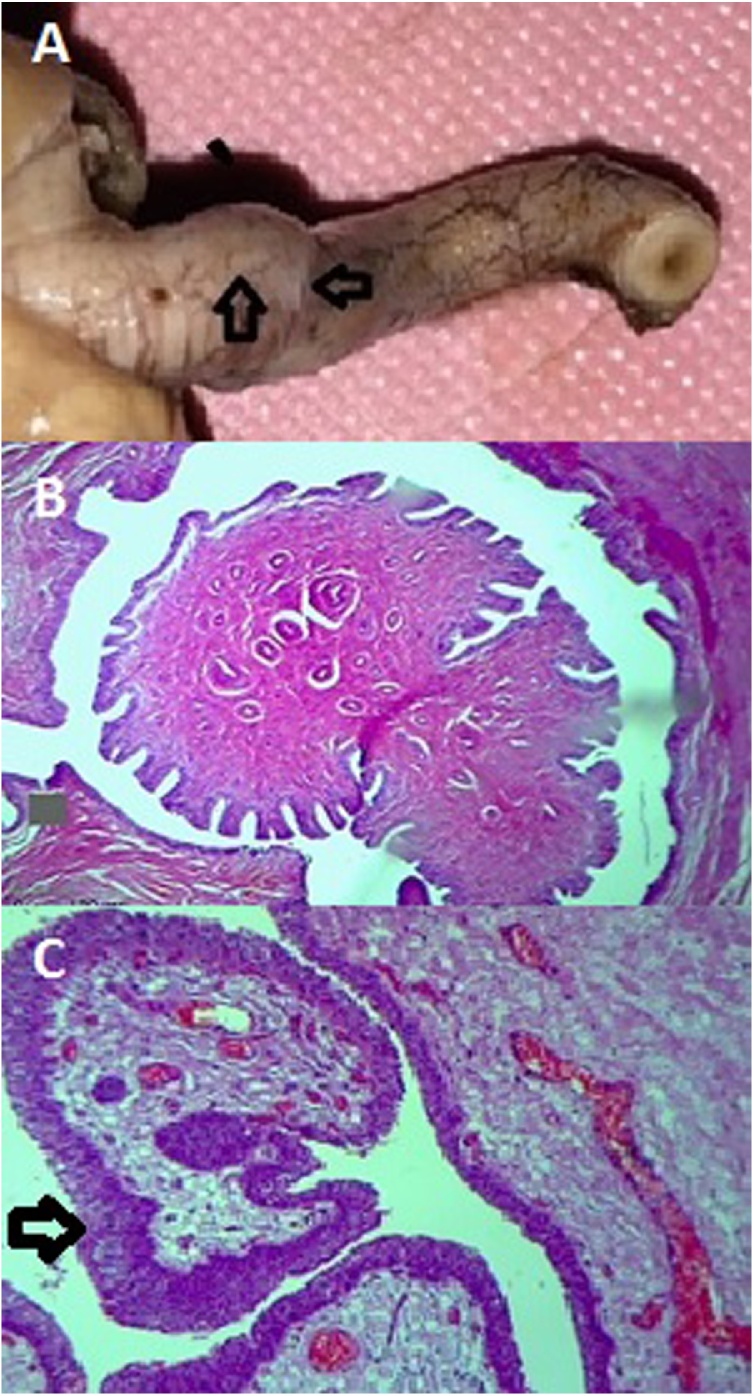
(A) Macroscopic study of specimen shows a segment of proximal ureter, measuring 4 cm in length that reveals a mass in the middle part of the ureter; (B) HE staining showing a papillomatous tumor; (C) Papillae show slender with minimal branching composed of a delicate fibrovascular core with numerous small capillaries which covered by proliferating normal-appearing urothelium lacking atypia.

A 13-year-old boy presented in July 2019 with right flank pain and intermittent macroscopic hematuria for four months. On past medical history, there are no records of trauma, renal disease, or renal stone. During the examination, the pulse rate 80 beats per minute with a regular rhythm, and the blood pressure was 120/70 mmHg. On physical examination, the patient's vital signs were stable. The patient mentioned a feeling of pain and tenderness in the right flank. No other reportable hints represented on examination. The ultrasonography investigation identified a 1.5 cm heterogeneous hyperechogenic ipsilateral lesion located in the upper segment of left ureter emerging near the ureteropelvic junction. Ultrasonography shows dilation of the right ureter and renal pelvis with moderate hydronephrosis observed (Fig. 1A). The retrograde urography confirmed a 1.5 cm lesion with filling defects in the proximal segment of the right ureter (Fig. 1B). Excretory phase image from an abdominal computed tomography scan demonstrated moderate dilation of the right renal pelvis and calyces, with related cortical atrophy due to hydronephrosis (Fig. 1C). Proximal ureterectomy was done through the right flank incision and sent for histopathological. The formalin-fixed, paraffin-embedded tissue prepared. On the macroscopic study of the specimen, after a longitudinal incision, a sessile mass with a defined margin with 1.5 cm in diameter was exposed in the middle part of the sample was extended along the ureter (Fig. 2A). On histologic examination, there was a papillomatous tumor. Papillae show slender with minimal branching composed of a delicate fibrovascular core with numerous small capillaries which covered by proliferating normal-appearing urothelium lacking atypia (Fig. 2B), including the presence of umbrella cells (Fig. 2C). The pathologic diagnosis was urothelial papilloma, with no evidence of malignant change. The patient had an uneventful recovery with no voice changes. The patient's health as well and the patient's symptoms disappeared. The patient discharged after 3 days and 1 low-pressure suction drains remained in place for 2 days. Follow up done for 2 months with no reported complications. The patient is now in good health.

## 3 Discussion

Overall, primary benign neoplasms of the ureters are evidenced by the few instances in the medical literature. Approximately 1 percent of the tumors observed in the upper urinary tract are primary tumors of the ureter. About 250 cases of primary ureteral tumors have reported in the literature [11]. The ureter, like other structures of the urinary tract, could develop neoplasms of epithelial origin. The differential diagnosis of the rarity of the benign urothelial tumor is critical [12,13]. Urothelial papilloma is sporadic in children, and macroscopic hematuria is the essential presenting symptom [14]. Jorge Isaac et al. reported a case of inverted papilloma of the urinary bladder in an 11-year-old. Clinical presentation of their evidence was macroscopic hematuria [15]. Kamarulzaman et al. presented a case of inverted papilloma of the urinary bladder in a 12-year-old girl who complained of intermittent hematuria. The initial ultrasound showed the presence of bladder mass. All clinical signs of patient resolved after endoscopic resection [16]. We present the second case of ureteral papilloma in a 13-year-old boy. Previously, the first case reported by Bocconi-Gibod.

## 4 Conclusion

Since primary urothelial papilloma of the ureter is rare in children, the Clinical findings and biologic implications of the tumor are still obscure. Therefore, the risks of local recurrence and progression of this lesion are still uncertain. The study, in more cases and long term clinical follow-up, give us a chance to understand the best diagnostic and treatment methods.

## Declaration of Competing Interest

None.

## Funding

The research did not receive any funding.

## Ethical approval

This case report was approved by the Research Ethics Committee of the Ali asghar pediatric Hospital.

## Consent

Written informed consent was obtained from the parents of patient for publication of this case report.

## Author contribution

Maryam Rahbar: described in the case report, concept and design of study, acquisition of data, drafting the manuscript, revising the manuscript, and approving the final version of the manuscript.Mahtab Rahbar and Seyed Javad Nasiri: described in the case report, revising the manuscript, and approving the final version of the manuscript.

## Registration of research studies

Not applicable.

## Guarantor

Mahtab rahbar.

## Provenance and peer review

Not commissioned, externally peer-reviewed.
